# Design, Synthesis, and Antitumor Evaluation of Novel Pyrazolo[3,4-*d*]pyrimidine Derivatives

**DOI:** 10.3797/scipharm.1204-23

**Published:** 2012-06-25

**Authors:** Manal M. Kandeel, Lamia W. Mohamed, Mohammed K. Abd El Hamid, Ahmed T. Negmeldin

**Affiliations:** Pharmaceutical Organic Chemistry Department, Faculty of Pharmacy, Cairo University, Cairo, Egypt.

**Keywords:** Pyrazolopyrimidines, Antitumor Activity, Cytotoxic Activity, Synthesis

## Abstract

A new series of pyrazolo[3,4-*d*]pyrimidines has been synthesized. The new compounds were tested for their antitumor activity on 60 different cell lines, and some of the compounds were found to have potent antitumor activity. In particular, 2-hydroxybenzaldehyde [1-(4-chlorophenyl)-3-methyl-1*H*-pyrazolo-[3,4-*d*]pyrimidin-4-yl]hydrazone (**VIIa**) was found to be the most effective among the other derivatives, showing IC50 values of 0.326 to 4.31 μM on 57 different cell lines.

## Introduction

**Fig. 1:**
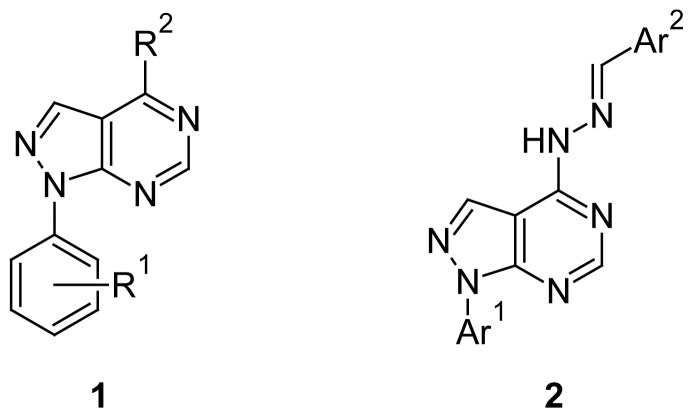
Structures of some reported antitumor pyrazolo[3,4-*d*]pyrimidines

Increasing interest in biological studies of pyrazolo[3,4-*d*]pyrimidines in the last decade is a consequence of their wide usage as a pharmaceutically important class of compounds [1]. Pyrazolopyrimidine derivatives have considerable potential in the field of chemotherapy, as they were found to exhibit their antitumor activity by inhibiting different types of enzymes such as cyclin-dependent kinase [2–4], Src and Abl tyrosine kinase [5], glycogen synthase kinase-3 [6–8], adenosine deaminase [9], and epidermal growth factor receptor protein tyrosine kinase [10]. The derivatives of pyrazolo[3,4-*d*]pyrimidine have already been discovered as antitumor agents by the NCI (National Cancer Institute, USA) on HCT116 and other cell lines. The potency of these compounds is enhanced in anilide derivatives, and this translates into tumor growth inhibition in a mouse xenograft model [2]. Some pyrazolo[3,4-*d*]pyrimidines (**1**, Figure 1) structurally related with allopurinol, have also been reported as potent inhibitors of xanthine oxidase and the growth of several human tumor cell lines [11]. In addition, several substituted pyrazolo[3,4-*d*]pyrimidines (**2**) were reported as potent antitumor agents [12].

Both the above findings and 4-substituted-1*H*-pyrazolo[3,4-*d*]-pyrimidines were reported to be cytotoxic and antitumor agents [1, 13–15]. In order to explore this possibility, compounds were prepared that had diverse groups at position 4 of the pyrazolopyrimidine core, and their antitumor activity was tested.

## Results and Discussion

### Chemistry

The synthesis of the designed compounds is outlined in schemes 1, 2, and 3. The main precursors for the synthesis of target derivatives, i.e. **Ia,b** and **IIa–c**, were prepared by a previously published synthetic method presented in Scheme 1. Compounds **IIIa,b** were prepared according to the synthetic methods presented in Scheme 2, either by heating compounds **Ia,b** in formamide or by heating compounds **IIa,b** in formic acid. Both procedures had high yields, but the second one had an even higher yield. Compound **IIIc** was prepared only by using one synthetic method from derivative **IIc** in formic acid, as it had the better yield. Structures of newly prepared compounds were confirmed by ^1^H NMR, IR, mass spectroscopy, and microanalyses.

On the other hand, synthesis of **IVa–c** presented in scheme 2 was performed by the reflux of **IIIa–c** in phosphorus oxychloride. The structures of the new compounds were confirmed by ^1^H NMR, which revealed disappearance of the singlet D_2_O exchangeable signal corresponding to NH, and showed an increased deshielding of H at position 6, due to the inductive effect of the chlorine atom. It was also confirmed by mass spectra which gave fragments showing the isotopic pattern of the chlorine atom.

The synthesis of **Va–g** and **VIIa–c** was outlined in scheme 3. First, **Va–g** were obtained by the reflux of **IVa–c** with the appropriate amine using triethylamine as a catalyst, and the formed derivatives were confirmed by ^1^H NMR, which revealed appearance of the singlet D_2_O exchangeable signal corresponding to NH, and appearance of other signals characterizing the introduced groups. The structures of some of these derivatives were additionally confirmed by mass spectra. ^13^C NMR was performed on compounds **Va**, **Vc**, and **VIIa. Va** showed distinct aromatic carbons where the carbon at position 3 of the pyrazole ring appeared at 101.76, while that of the pyrimidine ring at position 6 was the highest desheilded and appeared at 156.35ppm. On the other hand, the aromatic ring at position 1 of the pyrazole ring gives aromatic carbons, which are given the numbers 1′,2′,3′,4′,5′, and 6′, and that attached to the amine at the pyrimidine ring gives aromatic carbons marked as 1″, 2″, 3″, 4″, 5″ and 6″. **Vc** showed two distinct doublet signals belonging to the two CH_2_ groups attached to the NH group. The presence of the ethylene spacer was further confirmed by DEPT technique.

Second, **VIIa-c** were synthesized by the reflux of **IVc** with hydrazine, producing **VI** which was confirmed by ^1^H NMR, IR spectrum, microanalysis, and mass spectrum in which appearance of a singlet proton at δ 8.36 ppm in ^1^H NMR indicated slight shielding of the proton at position 6 of the pyrimidine ring, due to the removal of the chlorine atom, and a broad range of the D_2_O exchangeable signal of NH and NH_2_ groups at 3.82 and 4.95 ppm, and by the mass spectrum which shows M^+^, M^+^ + 2 at m/z 274, 276 as 100.00%, 49.15%.

Furthermore, **VI** was condensed with appropriate aldehydes producing **VIIa–c,** the structure of which was confirmed by ^1^H NMR, ^13^C NMR, IR spectra, microanalyses, and mass spectra. The IR spectrum shows the disappearance of the IR peak equivalent to NH_2_. In ^1^H NMR, the appearance of the D_2_O exchangeable broad singlet indicated the presence of NH, deshielded singlet of N=CH, in addition to different peaks equivalent to different substituents in each derivative. The OH group of compound **VIIa** appeared in the IR at 3369 cm^−1^ as an exchangeable peak in ^1^H NMR at10.20ppm. The OCH_3_ group of compound **VIIb** was detected by ^1^H NMR appearing more deshielded at 3.82 ppm than the CH_3_ group at position 3 at 2.79 ppm. Compound **VIIc** mainly showed the NO_2_ group in the IR at 1437, 1346 and the aromatic protons of the benzene ring 2″, 4″, 5″, and 6″ with a deshielded singlet signal equivalent to 2″ at 8.7–8.8, proving the m-substitution.

### Antitumor activity

The antitumor activity was determined for the newly synthesized compounds at the NCI for *in vitro* one-dose testing and detection of IC50 of their antitumor activity on 60 different cell lines. Compound **Vc**, **Vg**, **VIIa**, and **VIIc** were found to have the highest inhibitory activity on many cell lines. The obtained results of the tested derivatives showed a distinctive potential pattern of selectivity, as well as broad-spectrum antitumor activity (Table 1). Compound **VIIa** was subjected to 5-dose testing, as it showed the highest activity among other derivatives showing inhibition of 57 different cell lines (Table 2).

## Experimental

### Chemistry

All melting points were determined on the Stuart apparatus and the values given are uncorrected. The IR spectra were determined on the Shimadzu IR 435 spectrophotometer (KBr, cm^−1^), Faculty of Pharmacy, Cairo University, Egypt. The ^1^H NMR and ^13^C NMR spectra were recorded on the Varian Gemini 75 MHz spectrophotometer using TMS as the internal standard. Chemical shift values are recorded in ppm on the δ scale, Microanalysis Center, Cairo University, Egypt. Mass spectra were recorded on a Hewlett Packard 5988 spectrometer, Microanalysis Center, Cairo University, Egypt. Elemental analyses were carried out at the Microanalysis Center, Cairo University, Egypt; the values found were within ±0.35% of the theoretical ones. Progress of the reactions was monitored using TLC sheets precoated with the UV fluorescent silica gel, Merck 60F 254, and were visualized using a UV lamp.

The synthesis of the target compounds is outlined in Schemes 1–3. Compounds **Ia** [16], **Ib** [17], **IIa–c** [18–20], **IIIa** [21], and **Iva** [22] were prepared as reported in the literature.

### Procedures for the synthesis of compounds IIIb and IIIc

#### Method 1

A suspension of the appropriate derivative **Ia,b** (0.01 mol) in formamide (30 ml) was stirred at 145°C for 3 h; the solution was then cooled by being poured on ice-cold water, filtered, washed with water, dried, and finally crystallized from formic acid.

#### Method 2

A suspension of the appropriate derivative **IIa–c** (0.01 mol) in 85% formic acid (40 ml), was heated under reflux for 7 h; the reaction mixture was then cooled, filtered, washed with water, dried, and crystallized from formic acid.

#### 3-Methyl-1-(4-nitrophenyl)-1,5-dihydro-4H-pyrazolo[3,4-d]pyrimidin-4-one (**IIIb**)

Yield: 2.6 g (96% by method 2); M.p.: >300°C; ^1^H NMR (300 MHz, DMSO-d_6_): δ = 3.15 (s, 3H, CH_3_); 8.20 (s, 1H, C6-H); 8.34 (d, *J =* 9.3 Hz, 2H, ArH C2’,6’); 8.39 (d, *J =* 9.6 Hz, 2H, ArH C3’,5’); 12.51 (s, 1H, NH, D_2_O exchangeable) ppm; IR (cm^−1^): 3370(NH), 3075, 3034 (CH aromatic), 2911 (CH aliphatic),1686 (C=O), 1589 (C=N),1441,1337(NO_2_); MS (70 ev): *m/z* 271 (M^+^, 100%). Anal. Calcd for C_12_H_9_ N_5_O_3_ (271.23): C, 53.14; H, 3.34; N, 25.82. Found: C, 53.30; H, 3.64; N, 26.36

#### 1-(4-Chlorophenyl)-3-methyl-1,5-dihydro-4H-pyrazolo[3,4-d]pyrimidin-4-one (**IIIc**)

Yield: 1.94 g (74% by method 2); M.p.: >300°C; ^1^H NMR (300 MHz, DMSO-d_6_): *δ* = 3.30 (s, 3H, CH_3_); 7.60 (d, *J =* 9.0 Hz, 2H, ArH C3’,5’); 8.08 (d, 2H, *J =* 9.0 Hz, ArH C2’,6’); 8.15 (s, 1H, C6-H); 12.37 (s, 1H, NH, D2O exchangeable) ppm; IR (cm^−1^): 3422 (NH), 3121, 3040 (CH aromatic) 2974 (CH aliphatic),1670 (C=O), 1589 (C=N); MS (70 ev): *m/z* 261 (M^+^ + 1, 9.24%). Anal. Calcd for C_12_H_9_ClN_4_O (260.68): C, 55.29; H, 3.48; N, 21.49. Found: C, 55.36; H, 3.69; N, 21.41.

### General procedure for the synthesis of compounds IVb and IVc

A suspension of the appropriate derivative **IIIa–c** (0.01 mol) in phosphorus oxychloride (80 ml) was heated under reflux for 3 h; the solution was cooled and then poured onto ice-cold water. The precipitated product was filtered, dried, and crystallized from ethanol.

#### 4-Chloro-3-methyl-1-(4-nitrophenyl)-1H-pyrazolo[3,4-d]pyrimidine (**IVb**)

Yield: 2.03 g (70%), M.p.: 210–212°C; ^1^H NMR (200 MHz, DMSO-d_6_): *δ* = 2.71 (s, 3H, CH_3_); 8.37 (d, *J =* 9.4 Hz, 2H, ArH C2’,6’); 8.41 (d, *J =* 9.4 Hz, 2H, ArH C3’,5’); 8.96 (s, 1H, C6-H) ppm; IR (cm^−1^): 3120, 3080 (CH aromatic), 2905 (CH aliphatic), 1597,1576 (C=N), 1445, 1341 (NO_2_); MS (70 ev): *m/z* 289 (M^+^, 100%). Anal. Calcd for C_12_H_8_ClN_5_O_2_ (289.68): C, 49.75; H, 2.78; N, 24.18. Found: C, 49.60; H, 2.90; N, 24.22.

#### 4-Chloro-1-(4-chlorophenyl)-3-methyl-1H-pyrazolo[3,4-d]pyrimidine (**IVc**)

Yield: 1.48 g (53%), M.p.: 189–190°C; ^1^H NMR (300 MHz, DMSO-d_6_): *δ* = 3.40 (s, 3H, CH_3_); 7.65 (d, *J =* 9.0 Hz, 2H, ArH C3’,5’); 8.16 (d, 2H, *J =* 9.0 Hz, ArH C2’,6’); 8.92 (s, 1H, C6-H) ppm; IR (cm^−1^): 3095, 3065 (CH aromatic), 2924 (CH aliphatic), 1589,1574 (C= N); MS (70 ev): *m/z* 278 (M^+^, 87.22). Anal. Calcd for C_12_H_8_Cl_2_N_4_ (279.12): C, 51.64; H, 2.89; N, 20.07. Found: C, 51.43; H, 3.01; N, 19.81.

### General procedure for the synthesis of compounds Va–g

A suspension of the appropriate derivative **IVa–c** (0.01 mol) and the appropriate amine (0.01 mol) in ethanol (30 ml), triethylamine (0.3 g, 0.03 mol), was added and the reaction mixture was heated under reflux for 2–7 h; (the reaction was monitored using TLC until the starting materials were consumed in the reaction). The reaction mixture was allowed to cool leading to separation of the product, and then the crude product was filtered, dried, and crystallized from the appropriate solvent.

#### 3-Methyl-N,1-diphenyl-1H-pyrazolo[3,4-d]pyrimidin-4-amine (**Va**)

Yield: 0.15 g (49%), M.p.: 144–145°C (ethanol/water); ^1^H NMR (200 MHz, CDCl_3_): *δ* = 2.88 (s, 3H, CH_3_); 7.00 (s, 1H, NH); 7.20–7.35 (m, 3H, ArH C3”,4”,5”); 7.40–7.55 (m, 3H, ArH C3’,4’,5’); 7.72 (d, *J =* 9.0 Hz, 2H, ArH C2”,6”); 8.20 (d, *J =* 9.0 Hz, 2H, ArH C2’,6’); 8.55 (s, 1H, C6-H) ppm.; ^13^C NMR (75 MHz, CDCl_3_): *δ* = 15.17 (q, 1C, CH_3_); 101.76 (s, 1C, C 3a); 121.48 (d, 2C, ArC 2”,6”); 121.82 (d, 2C, ArC 2’,6’); 124.84 (d, 1C, ArC 4”); 126.38 (d, 1C, ArC 4’); 129.15 (d, 2C, ArC 3’,5’); 129.22 (d, 2C, ArC 3”,5”); 137.73 (s, 1C, ArC 1’); 138.73 (s, 1C, ArC 1”); 140.73 (s, 1C, C 3); 154.30 (s, 1C, C 7a); 155.60 (s, 1C, C 4); 156.35 (d, 1C, C 6) ppm; IR (cm^−1^): 3455 (NH), 3080, 3020 (CH aromatic), 2930 (CH aliphatic); MS (70 ev): *m/z* 301 (M^+^, 78.78%). Anal. Calcd for C_18_H_15_N_5_ (301.35): C, 71.74; H, 5.02; N, 23.24. Found: C, 72.00; H, 4.97; N, 23.07.

#### N-(4-Methoxyphenyl)-3-methyl-1-phenyl-1H-pyrazolo[3,4-d]pyrimidin-4-amine (**Vb**)

Yield: 0.20 g (59%), M.p.: 121–122°C (ethanol/water); ^1^H NMR (200 MHz, DMSO-d_6_): *δ* = 2.77 (s, 3H, CH_3_); 3.80 (s, 3H, OCH_3_); 6.99 (d, *J =* 6.6 Hz, 2H, ArH C3”,5”); 7.32 (t, *J =* 6.6 Hz, 1H, ArH C4’); 7.55 (d, *J =* 6.0 Hz & m, 4H, ArH C2”,6”,3’,5’); 8.20 (d, *J =* 8.2 Hz, 2H, ArH C2’,6’); 8.38 (s, 1H, C6-H); 8.76 (s, 1H, NH, D2O exchangeable) ppm; IR (cm^−1^): 3439 (NH), 3080, 3028 (CH aromatic), 2976 (CH aliphatic). Anal. Calcd for C_19_H_17_N_5_O (331.37): C, 68.87; H, 5.17; N, 21.13. Found: C, 68.90; H, 5.00; N, 21.01

#### 3-Methyl-1-phenyl-N-(2-phenylethyl)-1H-pyrazolo[3,4-d]pyrimidin-4-amine (**Vc**)

Yield: 0.10 g (30%), M.p.: 151–152°C (ethanol/water); ^1^H NMR (200 MHz, CDCl_3_): *δ* = 2.45 (s, 3H, CH_3_); 3.05 (t, *J =* 9.0 Hz, 2H, CH_2_); 3.88 (t, *J =* 9.0 Hz, 2H, CH_2_); 5.20 (s, 1H, NH); 7.25–7.40 (m, 5H, ArH”); 7.45–7.55 (m, 3H, ArH C3’,4’,5’); 8.18 (d, *J =* 9.0 Hz, 2H, ArH C2’,6’); 8.47 (s, 1H, C6-H) ppm; ^13^C NMR (75 MHz, CDCl_3_): *δ* = 14.73 (q, 1C, CH_3_); 35.33 (t, 1C, CH_2_); 41.70 (t, 1C, CH_2_); 101.33 (s, 1C, ArC C 3a); 121.38 (d, 2C, ArC C 2’,6’); 126.14 (d, 2C,ArC C4’, C4”); 126.89 (d, 2C, ArC C2”,6”); 128.89 (d, 2C, ArC C3”,5”); 129.08 (d, 2C, ArC C3’,5’); 138.56 (s,1C, ArC C 1”); 138.89 (s, 1C, ArC C 1’); 141.17 (s, 1C, ArC C 3); 153.99 (s, 1C, ArC C7a); 156.66 (s, 1C, ArC C6); 157.53 (s, 1C, ArC C4) ppm; IR (cm^−1^): 3428 (NH), 3030 (CH aromatic), 2940, 2922 (CH aliphatic). MS (70 ev): *m/z* 329 (M^+^, 16.33%). Anal. Calcd for C_20_H_19_N_5_ (329.40): C, 72.93; H, 5.81; N, 21.26. Found: C, 72.73; H, 5.90; N, 21.11.

#### 3-Methyl-1-(4-nitrophenyl)-N-phenyl-1H-pyrazolo[3,4-d]pyrimidin-4-amine (**Vd**)

Yield: 0.28 g (80%), M.p.: 259–260°C (benzene); ^1^H NMR (300 MHz, DMSO-d_6_): *δ* = 2.78 (s, 3H, CH_3_); 7.20 (t, *J =* 7.2 Hz, 1H, ArH C4”); 7.40 (t, *J =* 7.8 Hz, 2H, ArH C3”,5”); 7.68 (d, *J =* 6.6 Hz, 2H, ArH C2”,6”); 8.40 (d, *J =* 7.2 Hz, 2H, ArH C2’,6’); 8.49 (s, 1H, C6-H); 8.58 (d, *J =* 7.2 Hz, 2H, ArH C3’,5’); 8.88 (s, 1H, NH, D2O exchangeable) ppm; IR (cm^−1^): 3431 (NH), 3040 (CH aromatic), 2900 (CH aliphatic), 1443, 1344 (NO_2_). Anal. Calcd for C_18_H_14_N_6_O_2_ (346.34): C, 62.42; H, 4.07; N, 24.27. Found: C, 62.59; H, 3.99; N, 24.57.

#### 3-Methyl-1-(4-nitrophenyl)-N-(2-phenylethyl)-1H-pyrazolo[3,4-d]pyrimidin-4-amine (**Ve**)

Yield: 0.13 g (36%), M.p.: 214°C (benzene); ^1^H NMR (200 MHz, DMSO-d_6_): *δ* = 2.63 (s, 3H, CH_3_); 2.96 (t, *J =* 7.0 Hz, 2H, CH_2_); 3.80 (t, *J =* 7.0 Hz, 2H, CH_2_); 7.20–7.39 (m, 5H, ArH”); 7.53 (s, 1H, NH, D2O exchangeable); 8.39 (d, *J =* 9.4 Hz, 2H, ArH C3’,5’); 8.45 (s, 1H, C6-H); 8.57 (d, *J =* 9.4 Hz, 2H, ArH C2’,6’) ppm; IR (cm^−1^): 3439 (NH), 3080, 30210 (CH aromatic), 2940, 2920 (CH aliphatic), 1441, 1346 (NO_2_).; MS (70 ev): *m/z* 374 (M^+^, 13.47%). Anal. Calcd for C_20_H_18_N_6_O_2_ (374.4): C, 64.16; H, 4.85; N, 22.45. Found: C, 64.27; H, 4.89; N, 22.66.

#### 1-(4-Chlorophenyl)-N-(4-methoxyphenyl)-3-methyl-1H-pyrazolo[3,4-d]pyrimidin-4-amine (**Vf**)

Yield: 0.26 g (70%), M.p.: 198–200°C (ethanol); ^1^H NMR (300 MHz, DMSO-d_6_): *δ* = 2.74 (s, 3H, CH_3_); 3.78 (s, 3H, OCH_3_); 6.97 (d, *J =* 7.2 Hz, 2H, ArH C3”,5”); 7.53 (d, *J =* 7.0 Hz, 2H, ArH C2”,6”); 7.58 (d, *J =* 6.6 Hz, 2H, ArH C3’,5’); 8.25 (d, *J =* 6.9 Hz, 2H, ArH C2’,6’); 8.36 (s, 1H, C6-H); 8.74 (s, 1H, NH, D_2_O exchangeable) ppm; IR (cm^−1^): 3443 (NH), 3010 (CH aromatic), 2934, 2915 (CH aliphatic); MS (70 ev): *m/z* 365 (M^+^, 24.61%). Anal. Calcd for C_19_H_16_ClN_5_O (365.82): C, 62.38; H, 4.41; N, 19.14. Found: C, 65.59; H, 4.43; N, 18.95.

#### 1-(4-Chlorophenyl)-3-methyl-N-(2-phenylethyl)-1H-pyrazolo[3,4-d]pyrimidin-4-amine (**Vg**)

Yield: 0.24 g (65%), M.p.: 123–125°C (ethanol/water); ^1^H NMR (300 MHz, DMSO-d_6_): *δ* = 2.61 (s, 3H, CH_3_); 2.95 (t, *J =* 7.8 Hz, 2H, CH_2_); 3.76 (t, *J =* 7.5 Hz, 2H, CH_2_); 7.21–7.34 (m, 5H, ArH”); 7.34 (s, 1H, NH, D_2_O exchangeable); 7.56 (d, *J =* 9.0 Hz, 2H, ArH C3’,5’); 8.24 (d, *J =* 9.0 Hz, 2H, ArH C2’,6’); 8.39 (s, 1H, C6-H) ppm; IR (cm^−1^): 3418 (NH), 3100, 3026 (CH aromatic), 2924, 2860 (CH aliphatic). Anal. Calcd for C_20_H_18_ClN_5_ (363.84): C, 66.02; H, 4.99; N, 19.25. Found: C, 66.13; H, 5.15; N, 19.19.

### Synthesis of 1-(4-chlorophenyl)-4-hydrazino-3-methyl-1H-pyrazolo[3,4-d]pyrimidine (VI)

Hydrazine hydrate (0.1 mol) was added to a suspension of **IVc** (0.01 mol) in ethanol (35 ml), the reaction mixture was heated under reflux for 2.5 h; the precipitated product was filtered, washed with ethanol, dried, and crystallized from ethanol.

Yield: 2.55 g (93%), M.p.: 244–246°C; ^1^H NMR (300 MHz, DMSO-d_6_): *δ* = 2.62 (s, 3H, CH_3_); 3.82 (s, 1H, NH, D_2_O exchangeable); 4.95 (broad s, 2H, NH_2_, D_2_O exchangeable); 7.56 (d, *J* = 9.0 Hz, 2H, H3’,5’); 8.22 (d, *J* = 9.0 Hz, 2H, H2’,6’); 8.36 (s, 1H, H6) ppm; IR (cm^−1^): 3294 (NH), 3278 & 3199 (NH_2_); MS (70 ev): *m/z* 276 (M^+^ + 2); 274 (100%) (M^+^). Anal. Calcd for C_12_H_11_ClN_6_ (274.71): C, 52.47; H, 4.04; N, 30.59. Found: C, 52.67; H, 4.24; N, 30.59.

### General procedure for the synthesis of compounds VIIa–c

A suspension of **VI** (1 mmol), and the appropriate aldehyde (1 mmol) in ethanol (35 ml), was heated under reflux with stirring for 3–4 h; the reaction mixture was then cooled and the separated precipitate was filtered, dried, and crystallized from ethanol.

#### 2-({2-[1-(4-Chlorophenyl)-3-methyl-1H-pyrazolo[3,4-d]pyrimidin-4-yl]hydrazinylidene}-methyl)phenol (**VIIa**)

Yield: 0.24 g (63%), M.p.: 269–270°C; ^1^H NMR (300 MHz, DMSO-d_6_): *δ* = 2.76 (s, 3H, CH_3_); 6.88–6.95 (m, 1H, H5”); 7.28–7.33 (m, 1H, H3”); 7.39–7.43 (m, 1H, H4”); 7.58–7.63 (m, 1H, H6”); 7.80 (d, *J* = 9.0 Hz, 2H, H3’,5’); 8.04 (d, *J* = 9.0 Hz, 2H, H2’,6’); 8.23 (s, 1H, HC=N); 8.60 (s, 1H, H6); 10.20 (broad s, 1H, OH, D_2_O exchangeable); 11.90 (broad s, 1H, NH, D_2_O exchangeable) ppm; ^13^C NMR (75 MHz, DMSO-d_6_): *δ* = 14.10 (q, 1C, CH_3_); 102.00 (s, 1C, ArC C3a); 117.80 (s,1C, ArC C 3”); 119.80 (d, 2C, ArC C2’,6’); 125.38 (s,1C, ArC C5”); 127.80 (s,1C, ArC C6”); 129.00 (d,2C, ArC C3’,5’); 131.50 (s,1C, ArC C4’); 132.90 (s,1C, ArC C 4”); 136.54 (s, 1C, ArC C 1”); 138.26 (s,1C, ArC C 1’); 144.93 (s, 1C,C=N); 147.85 (s, 1C, ArC C3); 149.50 (s, 1C, ArC C7a); 153.57 (s, 1C, ArC C6); 157.47 (s, 1C, ArC C2”); 157.71 (s, 1C, ArC C4) ppm; IR (cm^−1^): 3433 (NH), 3369 (OH); MS (70 ev): *m/z* 380 (M^+^ + 2); 378 (M^+^); 243 (100%). Anal. Calcd for C_19_H_15_ClN_6_O (378.82): C, 60.24; H, 3.99; N, 22.19. Found: C, 60.50; H, 3.89; N, 22.44.

#### 1-(4-Chlorophenyl)-4-[2-(4-methoxybenzylidene)hydrazinyl]-3-methyl-1H- pyrazolo[3,4-d]pyrimidine (**VIIb**)

Yield: 0.32 g (82%), M.p.: 247–248°C; ^1^H NMR (300 MHz, DMSO-d_6_): *δ* = 2.79 (s, 3H, CH_3_); 3.82 (s, 3H, OCH_3_); 7.00 (d, *J* = 8.7 Hz, 2H, H3”,5”); 7.60 (d, *J* = 9.0 Hz, 2H, H3’,5’); 7.65 (d, *J* = 8.7 Hz, 2H, H2”,6”); 7.95 (d, *J* = 9.0 Hz, 2H, H2’,6’); 8.20 (s, 1H, HC=N); 8.35 (s, 1H, H6); 11.87 (broad s, 1H, NH, D_2_O exchangeable) ppm; IR (cm^−1^): 3220 (NH); MS (70 ev): *m/z* 393 (M^+^ + 1); 392 (100%). Anal. Calcd for C_20_H_17_ClN_6_O (392.84): C, 61.15; H, 4.36; N, 21.39. Found: C, 61.39; H, 4.51; N, 21.10.

#### 1-(4-Chlorophenyl)-3-methyl-4-[2-(2-nitrobenzylidene)hydrazinyl]-1H- pyrazolo[3,4-d]pyrimidine (**VIIc**)

Yield: 0.35 g (86%), M.p.: 286–288°C; ^1^H NMR (300 MHz, DMSO-d_6_): *δ* = 2.77 (s, 3H, CH_3_); 7.56 (d, *J* = 9.0 Hz, 2H, H3’,5’); 7.78–7.84 (m, 2H, H5”,6”); 8.07 (d, *J* = 9.0 Hz, 2H, H2’,6’); 8.08–8.13 (m, 1H, H4”); 8.33 (s, 1H, HC=N); 8.53 (s, 1H, H6); 8.75–8.80 (m, 1H, H2”); 12.12 (broad s, 1H, NH, D_2_O exchangeable) ppm; IR (cm^−1^): 3327 (NH), 1437, 1346 (NO_2_). Anal. Calcd for C_19_H_14_ClN_7_O_2_ (407.81): C, 55.96; H, 3.46; N, 24.04. Found: C, 56.20; H, 3.56; N, 23.96.

### Antitumor activity

The compounds were prepared in DMSO: glycerol 9:1 at a concentration of 4 mM for the single-dose assay. The solution was diluted 1:400, giving a test concentration of 10 μM. The human tumor cell lines of the cancer screening panel were prepared according to the standard procedure of the American National Cancer Institute (NCI), and the tests were performed at the American National Cancer Institute (NCI) [23, 24].

Compound **VIIa**, which was subjected to a 5-dose assay, was prepared at a concentration 40 mM. The solution was diluted 1:400, giving a Test concentration of 100 μM.

**Fig. 2:**
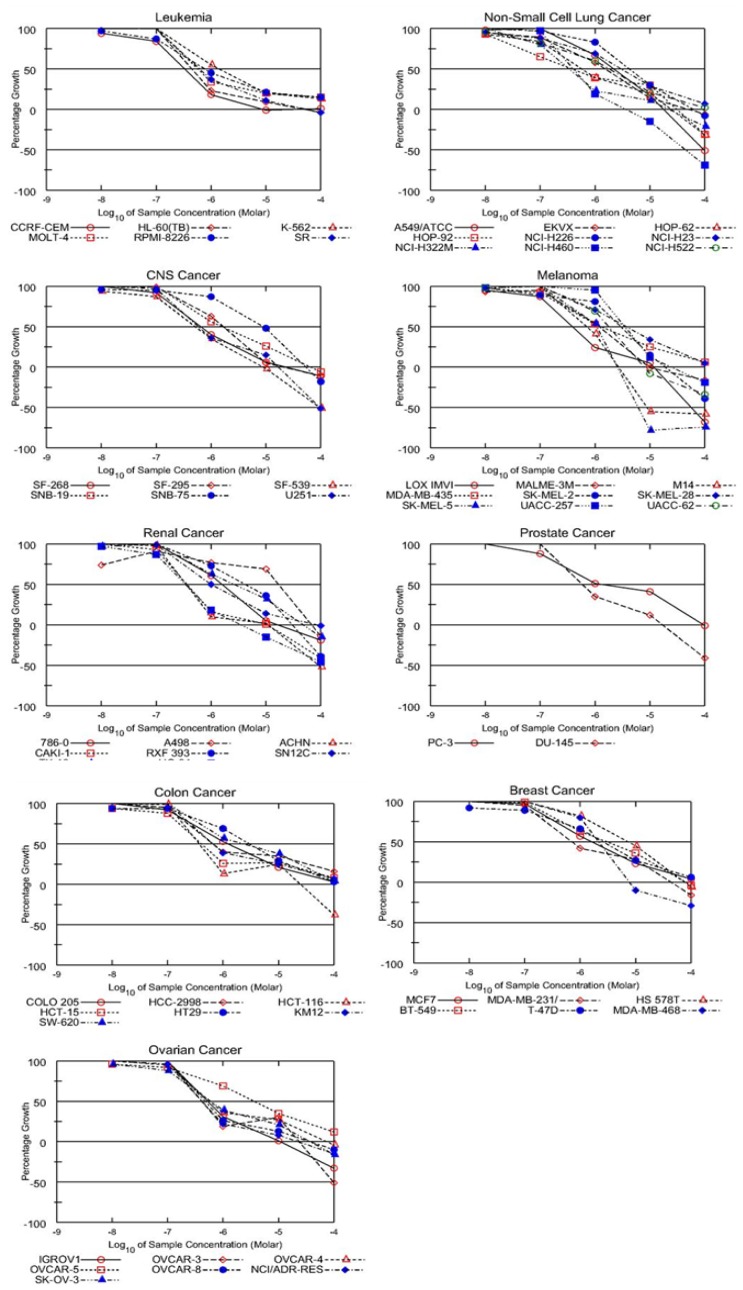
Dose response curves for compound **VIIa**.

The human tumor cell lines of the cancer screening panel were prepared according to the standard procedures of the NCI. The results are summarized in Tables 1 and 2, and Figure 2.

## Conclusion

The objective of this study was to synthesize and investigate the anticancer inhibition activity of selected pyrazolopyrimidines with the hope of discovering new structure leads to serve as potential anticancer agents. The newly synthesized compounds showed good inhibitory activity on different cell lines at a concentration of 10μM, making them leading chemical entities for further modification to render them as clinically useful therapeutic agents. Compound **Vc** showed 65.57% and 53.25% on the k-562 and molt-4 cell lines of leukemia. **Vg** produced 76.32 and 51.72 percentage inhibition on the K-562 and HOP-92 cell lines of leukemia and small lung cancer, **VIIc** showed 90% inhibition on the ovcar-3 cell line of ovarian cancer, and 82% and 84% inhibition on the k-562 and molt-4 cell lines of leukemia. The highest activity was presented by compound **VIIa**, which showed good inhibitory activity against 57 cell lines, and was the reason for performing 5-dose testing on this compound and measuring its GI50 (growth inhibition)and LC50 (lethal concentration), which further revealed its high potency against most of the cell lines.

## Figures and Tables

**Sch. 1:**
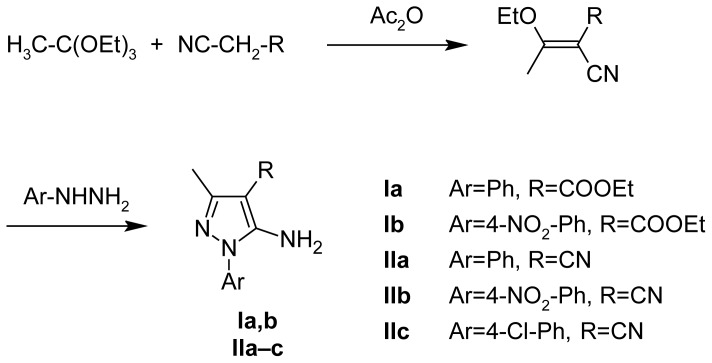
Synthesis of pyrazole intermediates

**Sch. 2:**
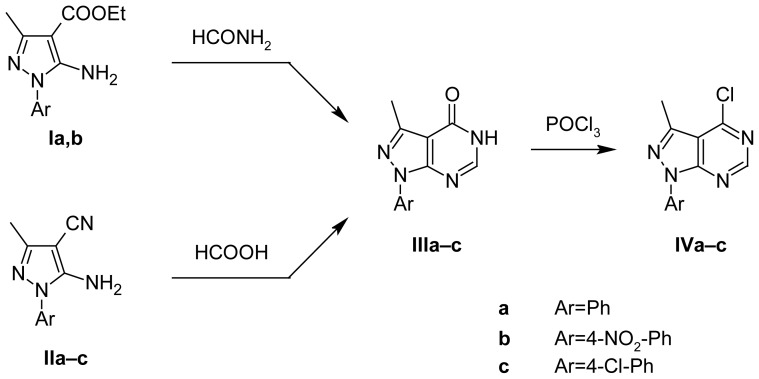
Synthesis of pyrazolo[3,4-*d*]pyrimidines

**Sch. 3:**
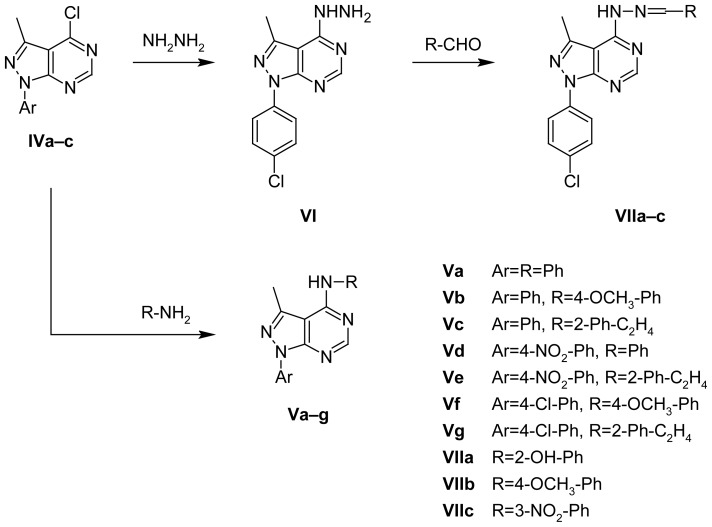
Synthesis of new pyrazolo[3,4-*d*]pyrimidine derivatives

**Tab. 1:** Inhibition percent of the tested compounds (10^−5^ Molar) on different 60 cell lines

Cell line type		Tested compounds and inhibition percent of cell lines									

		Va	Vb	Vc	Vd	Ve	Vf	Vg	VIIa	VIIb	VIIc
Leukemia	CCRF-CEM	19.43	4.00	6.94	2.25	–	2.45	0.33	**93.59**	–	**79.59**
	HL-60(TB)	29.20	11.40	22.22	8.87	35.22	–	29.98	**73.60**	–	**69.05**
	K-562	30.71	24.64	**65.57**	5.24	23.27	–	**76.32**	**81.45**	19.22	**82.62**
	MOLT-4	30.65	32.39	**53.25**	21.34	35.41	12.37	43.41	**90.57**	20.53	**84.60**
	RPMI-8226	23.57	9.63	12.33	16.39	3.17	19.90	17.27	**62.86**	6.07	33.66
	SR	36.93	41.72	**53.71**	28.86	37.77	20.94	34.91	**95.20**	18.44	**48.73**

Non-Small Cell Lung Cancer	A549/ATCC	–	4.05	6.18	10.26	0.26	3.61	–	**90.54**	–	–
	EKVX	–	5.68	8.75	–	**47.45**	13.84	19.40	**61.94**	–	15.99
	HOP-62	–	2.91	9.60	–	–	19.97	7.30	**92.07**	–	–
	HOP-92	34.14	8.10	11.46	6.51	8.78	28.98	**51.17**	**55.93**	12.52	**79.55**
	NCI-H226	–	–	–	–	2.27	10.33	3.81	**47.25**	–	–
	NCI-H23	–	–	–	–	4.22	10.44	4.32	**63.00**	–	5.70
	NCI-H322M	–	–	–	–	–	_	–	**66.66**	–	3.60
	NCI-H460	–	–	–	10.24	8.52	4.20	3.49	**94.96**	–	22.17
	NCI-H 522	8.39	9.21	17.72	19.25	–	28.80	4.48	_	–	–

Colon Cancer	COLO 205	4.67	–	–	–	–	8.75	6.22	**81.33**	–	4.75
	HCC-2998	–	–	–	–	–	–	_	**68.95**	–	5.17
	HCT-116	–	0.89	6.87	**33.53**	7.57	8.26	5.93	**73.26**	–	–
	HCT-15	3.74	9.57	7.60	–	4.73	6.88	6.10	**68.08**	–	–
	HT29	12.25	18.87	5.24	10.25	–	14.96	4.20	**79.92**	0.97	9.36
	KM12	–	–	–	–	4.92	5.48	–	**71.56**	–	33.45
	SW-620	0.79	–	–	–	0.72	2.59	–	**64.89**	–	11.24

CNS Cancer	SF-268	5.96	–	–	3.91	6.41	–	–	**90.33**	–	**41.99**
	SF-295	–	–	2.42	–	–	–	–	**75.51**	–	–
	SF-539	3.88	0.50	–	1.85	0.58	9.76	–	**85.97**	–	–
	SNB-19	–	–	–	2.95	2.93	1.78	–	**70.42**	–	3.66
	SNB-75	0.60	10.60	–	–	12,15	10.17	–	**36.41**	0.88	0.87
	U251	–	–	–	0.50	–	–	–	**86.92**	0.73	17.78

Melanoma	LOX IMVI	–	1.66	7.32	15.16	–	19.19	–	**64.88**	0.69	6.68
	MALME-3M	–	–	–	–	–	_	–	**51.30**	–	2.87
	MDAMB435	–	–	6.98	–	5.22	–	–	**72.73**	–	2.94
	SK-MEL-2	–	–	–	–	–	10.73	5.94	**87.67**	0.78	1.14
	SK-MEL-28	–	–	–	–	–	–	–	**74.82**	–	–
	SK-MEL-5	–	–	2.47	–	–	3.25	–	16.17	–	–
	UACC-257	–	–	–	–	–	–	–	**74.83**	–	–
	UACC-62	–	1.44	10.31	0.78	9.62	18.24	14.27	**86.54**	–	–

Ovarian Cancer	IGROV1	–	–	6.94	–	0.87	_	–	**76.08**	–	5.25
	OVCAR-3	–	–	–	–	–	–	–	**65.08**	–	**91.83**
	OVCAR-4	–	2.38	1.30	–	–	8.86	–	**80.50**	–	5.56
	OVCAR-5	5.33	15.68	8.84	–	–	4.24	7.88	**52.48**	–	–
	OVCAR-8	–	3.30	1.97	0.91	1.68	6.25	–	**85.94**	–	0.43
	NCI/ADR-RES	–	7.90	21.95	–	–	–	5.66	**80.90**	–	–
	SK-OV-3	–	–	–	–	–	24.57	7.19	**95.56**	–	–

Renal Cancer	786-0	3.00	8.93	11.46	–	–	–	–	**91.01**	–	3.71
	A498	18.12	12.14	7.44	–	18.37	–	–	21.46	8.64	14.62
	ACHN	0.89	6.27	5.86	–	0.28	13.55	7.13	**83.54**	–	–
	CAKI-1	–	–	23.73	–	12.87	—	29.75	**86.47**	4.83	24.71
	RXF 393	–	–	–	–	–	2.16	2.27	**74.27**	–	–
	SN12C	–	–	–	1.51	–	7.73	–	**76.94**	–	–
	TK-10	–	5.94	5.32	**70.00**	–	16.23	5.88	**83.60**	2.97	6.83
	UO-31	–	4.94	12.46	0.50	20.39	—	13.47	**64.77**	9.87	27.79

Prost.	PC-3	6.67	7.99	1.59	8.91	11.43	24.61	22.41	**58.41**	10.93	**61.01**
Canc.	DU-145	–	–	–	–	–	–	–	**80.91**	–	31.32

Breast Cancer	MCF7	–	4.97	–	6.68	6.98	15.10	1.80	**91.72**	4.60	–
	MDA-MB-231/ATCC	–	–	–	4.83	–	24.17	14.77	**67.43**	2.75	–
	HS578T	–	–	–	–	–	–	–	19.00	0.98	0.81
	BT-549	–	–	–	–	–	–	–	**63.98**	–	–
	T-47D	–	–	12.42	3.82	6.98	23.74	2.13	**86.73**	–	–
	MDA-MB-468	–	–	–	–	–	–	–	**47.74**	–	–

**Tab. 2:** Values detected from 5 dilutions for compound (**VIIa**) on 60 cell lines

Cell line type		GI50 (μM)	TGI (μM)	LC50 (μM)
Leukemia	CCRF-CEM	0.326	—	>100
	HL-60(TB)	0.522	48.6	>100
	K-562	1.37	>100	>100
	MOLT-4	0.625	>100	>100
	RPMI-8226	0.760	>100	>100
	SR	0.663	53.4	>100

Non-Small Cell Lung Cancer	A549/ATCC	2.22	17.8	95.7
	EKVX	1.50	52.0	>100
	HOP-62	0.559	24.0	>100
	HOP-92	0.383	30.9	>100
	NCI-H226	4.23	62.9	>100
	NCI-H23	2.97	>100	>100
	NCI-H322M	0.335	22.0	>100
	NCI-H460	0.398	3.61	45.0
	NCI-H 522	1.79	>100	>100

CNS Cancer	SF-268	0.646	23.0	>100
	SF-295	1.67	20.0	>100
	SF-539	0.522	8.84	96.9
	SNB-19	1.55	64.6	>100
	SNB-75	8.66	53.0	>100
	U251	0.595	16.9	95.1

Melanoma	LOX IMVI	0.389	11.9	57.1
	MALME-3M	1.19	9.56	>100
	M14	0.726	2.68	8.83
	MDAMB435	1.25	>100	>100
	SK-MEL-2	2.97	19.0	>100
	SK-MEL-28	3.70	>100	>100
	SK-MEL-5	1.06	2.55	6.11
	UACC-257	3.49	24.7	>100
	UACC-62	1.75	7.78	>100

Ovarian Cancer	IGROV1	0.546	10.7	>100
	OVCAR-3	0.427	23.5	97.1
	OVCAR-4	0.578	73.4	>100
	OVCAR-5	3.66	>100	>100
	OVCAR-8	0.477	36.1	>100
	NCI/ADR-RES	0.419	22.6	>100
	SK-OV-3	0.601	37.4	>100

Renal Cancer	786-0	1.58	15.8	>100
	A498	17.0	68.3	>100
	ACHN	0.350	10.8	92.9
	CAKI-1	0.360	10.5	>100
	RXF 393	4.31	30.7	>100
	SN12C	0.983	84.0	>100
	TK-10	2.60	46.9	>100
	UO-31	0.348	3.51	>100

Prostate	PC-3	1.36	92.3	>100
Cancer	DU-145	0.601	16.7	>100

Breast Cancer	MCF7	1.56	>100	>100
	MDA-MB-231/ATCC	0.719	42.7	>100
	HS578T	7.34	77.6	>100
	BT-549	3.05	78.3	>100
	T-47D	2.58	>100	>100
	MDA-MB-468	2.15	7.81	>100

Colon Cancer	COLO 205	1.27	>100	>100
	HCC-2998	0.656	>100	>100
	HCT-116	0.373	24.9	>100
	HCT-15	0.409	>100	>100
	HT29	2.94	>100	>100
	KM12	0.686	>100	>100
	SW-620	2.31	>100	>100
